# PReS-FINAL-2217: Atypical presentation of CRMO in two children

**DOI:** 10.1186/1546-0096-11-S2-P207

**Published:** 2013-12-05

**Authors:** F Vanoni, A Von Scheven-Gête, M Hofer

**Affiliations:** 1 DMCP-CHUV-Lausanne, Division of pediatric rheumatology, Lausanne, Switzerland

## Introduction

Chronic recurrent multifocal osteomyelitis (CRMO) is an autoinflammatory bone disorder that manifests as recurrent flares of inflammatory bone pain, related to one or more foci of nonbacterial osteomyelitis. Patients may present with low grade fever and modest elevation of the erythrocyte sedimentation rate (ESR), C-reactive protein (CRP) and white blood cell counts (WBC). We describe two cases of chronic recurrent multifocal osteomyelitis with high fever and pronounced elevation of inflammatory parameters.

## Methods

The first case was an 8-year-old boy presenting to the hospital with polyarticular pain, limping, fever, decreased appetite, weight loss and fatigue. CRP was 171 mg/l, ESR 81 mmh, WBC 16.5 G/l with 85% of neutrophils. We could exclude an infectious origin and suspected systemic onset juvenile idiopathic arthritis (SoJIA).

The patient responded well to NSAIDs, and after discontinuation he showed a stiff neck without history of trauma. Cervical MRI showed C2 and C4 vertebral compaction with bone oedema. Total body MRI showed right distal femoral, right distal fibular and left acetabular enhancement. CRMO was suspected and a fibular biopsy, performed to rule out a tumour, showed fibrous remodelling of the bone, supporting the diagnosis of CRMO. NSAIDs were restarted with progressive improvement. A follow up MRI 6 months later showed decrease of cervical vertebrae oedema.

The second case was a 7-year-old boy with 2 weeks of high fever, decreased appetite, and weight loss without perspiration or chills. Blood parameters showed CRP 105 mg/l, ESR 72 mm/h and thrombocytosis 651 G/l. WBC was normal. Infectious and onco-haematological origines were excluded. A few days later he complained about left wrist pain. An MRI showed a significant periosteal reaction of the two bones of the forearm, associated with soft tissue involvement. Bone scintigraphy revealed hypercaptation of both forearms suggesting CRMO. Bone biopsy showed no inflammation or other abnormalities.

Under NSAIDs, the patient did not improve and the biological parameters remained elevated. Because of the persistence of high fever and significant systemic inflammation a treatment with Anakinra, interleukin-1 (IL-1) receptor antagonist, was started, and induced rapid improvement of both bone pain and fever. When Anakinra was discontinued, inflammatory parameters increased again, without fever or other symptoms. A total body MRI was performed and showed multiple symmetrical enhancements in different skeletal segments.

## Conclusion

The two cases described showed an atypical presentation of CRMO, with high fever and increased inflammatory parameters suggesting a SoJIA. Interestingly, the second case responded to IL-1 blockade, suggesting a role for this cytokine in the disease. Another genetic syndrome, DIRA (deficiency of interleukin-1 receptor antagonist), presents with a similar phenotype to CRMO and could suggest an overlapping of these diseases with a key role of IL-1 in disease pathogenesis.

## Disclosure of interest

None declared.

